# *Orostachys japonicus* extract inhibits the lipopolysaccharide‐induced pro‐inflammatory factors by suppression of transcription factors

**DOI:** 10.1002/fsn3.1441

**Published:** 2020-02-24

**Authors:** Hyeong‐Seon Lee

**Affiliations:** ^1^ Department of Biomedical Laboratory Science Jungwon University Goesan South Korea

**Keywords:** AP‐1, cytokines, IRF‐3, *Orostachys japonicus*, pro‐inflammatory mediators

## Abstract

*Orostachys japonicus* (*O. japonicus*) was extracted with ethanol (EtOH) and sequentially separated with organic solvents, including n‐hexane (Hex), dichloromethane (DCM), ethyl acetate (EtOAc), n‐butanol (BuOH), and water (H_2_O). All the fractions were confirmed for anti‐inflammatory activity in an inflammatory condition. The DCM fraction showed the highest anti‐inflammatory ability. Here, we examined the effect of DCM fraction and investigated the intracellular signaling pathways in LPS‐stimulated RAW 264.7 macrophage cells. The DCM fraction significantly inhibited the mRNA levels of pro‐inflammatory mediators and cytokines including iNOS, COX‐2, IL‐1β, IL‐2, IL‐6, and IP‐10 in LPS‐stimulated cells. Also, the treatment of DCM fraction excellently reduced the expression of the proteins of AP‐1 (phospho‐c‐Jun and phospho‐c‐Fos) and phospho‐IRF3 as transcription factors. As a result, it suppressed LPS‐induced inflammatory mediator and cytokines via inhibition of transcription factors. In conclusion, our data demonstrated that DCM fraction has a strong anti‐inflammatory activity that improves the inflammatory state.

## Graphical abstract

We examined the effect of DCM fraction and investigated the intracellular signaling pathways in LPS‐stimulated RAW 264.7 macrophage cells. Our data demonstrated that DCM fraction has a strong anti‐inflammatory activity that improves the inflammatory state.

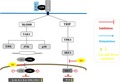

## 1 INTRODUCTION

Acute inflammation is an immediate immune response that occurs when cells are damaged by infection, injury, or stress (Lee et al., 2019). In addition, an excessive inflammatory response can cause chronic diseases, including arthritis, arteriosclerosis, and cancer (Grivennikov, Greten, & Karin, 2010; Mantovani & Pierotti, 2008). The macrophage in immune cells plays a central role in the immediate activation against antigenic substances. Lipopolysaccharide (LPS) is a typical composition of the wall of Gram‐negative bacteria cell. It is an endotoxin that stimulates phagocytes such as macrophage and neutrophil (Dou et al., 2013). Toll‐like receptor 4 (TLR4) on the surface of macrophage recognizes LPS and activates an intracellular signaling pathway (Qureshi et al., 2012). TLR4 is the starting point for the activation of two signaling pathways, such as the myeloid differentiation factor (MyD88)‐ and Toll/IL‐1R domain‐containing adaptor‐inducing interferon‐β (TRIF)‐mediated pathways (O'Neill, 2006). Stimulated MyD88 and TRIF activate translocation of nuclear factor‐κB (NF‐κB), activator protein 1 (AP‐1), and interferon regulatory factor 3 (IRF3), respectively (Fitzgerald et al., 2003). As a result, the activated macrophages release inflammatory factors and cytokines (Lee, Ryu, Lee, & Lee, 2013; Youn et al., 2006).

*Orostachys japonicus* is a polygamous plant in the family Crassulaceae that has been used as a medicinal herb for fever, hemostasis, antidote, inflammation, and cancer (Kim, Choi, Park, Lee, & Jung, 2009; Kwon & Han, 2004; Lee, Lee, Kim, Kim, et al., 2014; Lee, Ryu, et al., 2013; Lee, Lee, Kim, Suk, et al., 2014; Ryu, Lee, Lee, & Lee, 2012; Yoon et al., 2009). The previous studies have reported that *O. japonicus* has beneficial ingredients, such as flavonoids, triterpene, and galactic acid (Park, Han, Park, Choi, & Choi, 2005). Thus, dried *O. japonicus* was extracted with six kinds of organic solvents. The fractionated *O. japonicus* was expected to active various physiological processes, including inflammation. In this study, we confirmed the effective *O. japonicus* fraction for treating the LPS‐induced inflammatory condition. Furthermore, we wanted to find out the effect of *O. japonicus* on intracellular signals of cytokines and transcription factors in LPS‐stimulated macrophage cells.

## 2 MATERIALS AND METHODS

### 2.1 Cell culture and experimental reagents

Mouse macrophages (RAW 264.7 cells) were obtained from the Korean Cell Line Bank (KCLB). Cell was cultured on complete DMEM media added with 1% antibiotics (50× penicillin and streptomycin) and 10% fetal bovine serum (FBS) (Welgene) at 37°C in a 5% CO_2_. The macrophages were maintained to culture every 2–3 days at 1:6 split ratios. Rabbit primary antibodies against phospho‐c‐Jun, phospho‐c‐Fos, phospho‐IRF3, and GAPDH (housekeeping gene) were ordered from Cell Signaling Technology Inc.. HRP‐conjugated second antibody was purchased from BD Pharmingen™ (BD Biosciences).

### 2.2 Fractionated *Orostachys japonicus* with organic solvents

Fractionated *O. japonicus* was supplied from a farm in Miryang (Geobugiwasong Ltd.). The *O. japonicus* was separated using organic solvents, and the extract method was described in the previous studies (Lee, Lee, Kim, Kim, et al., 2014; Lee, Kim, & Lee, 2018; Lee, Lee, Kim, Suk, et al., 2014; Ryu et al., 2012). Each fraction removed the solvents by evaporator at 40°C to dryness. It was lysed in dimethyl sulfoxide (DMSO) and retained in a frozen state (Lee, Bilehal, et al., 2013).

### 2.3 Cell proliferation analysis

Cell viabilities were confirmed with an MTS assay kit (Promega Corporation) in the protocol. RAW 264.7 cells were incubated with serial doses (0, 25, 50, 75, and 100 μg/ml) of organic solvents for 24 hr. After the reaction, 20 μl of solution of cell proliferation assay was added and formed a formazan for 4 hr. The results were measured of absorbance at 490 nm using a FilterMax F5 microplate reader (Molecular Devices).

### 2.4 Reverse transcription polymerase chain reaction (RT‐PCR)

Cells were pretreated with solvent fractions of *O. japonicus* for 2 hr and then LPS‐induced inflammation for 12 hr. Total RNAs were separated with the cells of 6‐well plate using the Trizol™ reagent (Invitrogen). The concentrations of the total RNA were measured at 260 nm by a FilterMax F5 microplate reader (Molecular Devices); 2 μg RNA and 1 μg/μl oligo(DT) were added to AccuPower Reverse Transcription PreMix tube for the cDNA synthesis (Bioneer). The amplification of the target gene was performed using manufactured primers of forward and reverse in the PCR cycler. The primer sequences and conditions used in the PCR cycler are arranged in Table 1. After PCR, the products were transferred on 1.5% agarose gels and exposed the ethidium bromide (EtBr) in the electrophoresis system. The band density was determined and visualized using the Davinch‐Chemi™ imaging system (Davinch‐K).

**Table 1:** Primer sequence design for RT‐PCR

Target	Sequence of primers		Size of products (bp)
IL‐1β	Sense:	5′‐CAG GCA GGC AGT ATC ACT CA	350
	Antisense:	5′‐AGG CCA CAG GTA TTT TGT CG	
IL‐2	Sense:	5′‐CCC ACT TCA AGC TCC ACT TC	389
	Antisense:	5′‐TCC ACC ACA GTT GCT GAC TC	
IL‐6	Sense:	5′‐AGT TGC CTT CTT GGG ACT GA	159
	Antisense:	5′‐TCC ACG ATT TCC CAG AGA AC	
IP‐10	Sense:	5′‐GGA TGG CTG TCC TAG CTC TG	211
	Antisense:	5′‐ATA ACC CCT TGG GAA GAT GG	
iNOS	Sense:	5′‐GTG GTG ACA AGC ACA TTT GG	487
	Antisense:	5′‐GGC TGG ACT TTT CAC TCT GC	
COX‐2	Sense:	5′‐GCG AGC TAA GAG CTT CAG GA	498
	Antisense:	5′‐GAG AAG GCT TCC CAG CTT TT	
β‐action	Sense:	5′‐TGT TAC CAA CTG GGA CGA CA	392
	Antisense:	5′‐TCT CAG CTG TGG TGG TGA AG	

### 2.5 Protein expression analysis

After 1–2 hr of treatment, the cells were harvested by cell lysate buffer (Cell Signaling Technology) using a scraper (SPL Lifesciences) containing a protease inhibitor (Abcam plc.). The lysate was centrifuged to extract the protein from the supernatant and determined to concentrate the protein using a bicinchoninic acid (BCA) protein determination kit (Cayman Chemical); 40–50 μg of proteins was loaded to 8%–10% sodium dodecyl sulfate‐polyacrylamide gel electrophoresis (SDS‐PAGE) gel with running buffer. Semidry transfer system moved the proteins of gel to polyvinylidene fluoride (PVDF) membrane paper (Bio‐Rad). The membranes were incubated with 3% nonfat milk and reacted to specific antibodies for 18 hr at 4°C. The membrane was washed PBS and lastly treated with anti‐rabbit HRP‐conjugated second antibody for 2 hr at 4°C. The result levels on the membrane were developed with A and B ECL solution (Santa Cruz Biotechnology) and visualized by the Davinch‐Chemi^TM^ imaging system (Davinch‐K).

### 2.6 Statistical analysis

All experiments were carried out more than three times. The data were reported as the mean ± standard deviation (*SD*) and analyzed by SPSS (version 21). A difference between the experimental groups was significantly expressed at *p* < .05 and *p* < .01.

## 3 RESULTS AND DISCUSSION

In this study, the intracellular signaling mechanisms of the LPS‐stimulated inflammatory induction in RAW 264.7 macrophage cells were investigated. The *O. japonicus* was separated sequentially with organic solvents. These soluble fractions were examined for the effect of anticancer in various cells such as human gastric, hepatoma, colon, ovarian, and pancreatic cancer (Kim, Nam, Kim, Ryu, & Lee, 2019; Lee, Lee, Kim, Kim, et al., 2014; Lee et al., 2018; Lee, Lee, Kim, Suk, et al., 2014; Ryu, Lee, Kwon, & Lee, 2018; Ryu et al., 2012). Among these fractions, EtOAc and DCM extracts exhibited the highest effect for apoptosis signaling pathways. In addition, the anti‐inflammatory and antioxidant effects of *O. japonicus* have been verified (Lee, Bilehal, et al., 2013; Lee, Lee, Kim, Kim, et al., 2014; Lee, Lee, Kim, Suk, et al., 2014), and it will be applicable to the prevention and treatment of various diseases.

Among them, DCM fraction from *O. japonicus* (OJD) showed the best anti‐inflammatory effect on LPS‐stimulated cells. We used a gas chromatography‐mass spectrometry (GC‐MS) system to examine the active components in the DCM fraction. As a result, 11 peaks of them were hard to identify, but 3 peaks were identified as kaempferol (7.76%), quercetin (6.51%), and campesterol (53.53%) (Lee, Ryu, et al., 2013). Furthermore, research on the ingredients of *O. japonicus* will continue.

### 3.1 Effect of the *Orostachys japonicus* solvent fractions on cell viability

**Figure 1:**
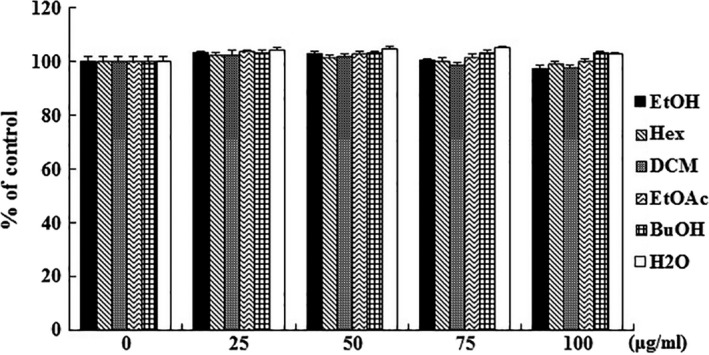
Cell proliferation effect of *Orostachys japonicus* solvent fractions in RAW 264.7 cells. Macrophage cells were incubated with solvent fractions (0, 25, 50, 75, and 100 μg/ml) for 24 hr, and the cell proliferation was assayed by MTS solution. The results are expressed as the mean ± *SD*

To assess the cytotoxic effect of the solvent fractions, the cells (5 × 10^5^/ml) were reacted to serial doses (0, 25, 50, 75, and 100 μg/ml) for 24 hr. As a result, the survival rate of cells exceeded 90% at all concentrations of the solvent fractions. Cell proliferation was not affected by any of the conditions. Based on this, the highest concentration was selected to be 100 μg/ml (Figure 1).

### 3.2 Effect of the *Orostachys japonicus* solvent fractions on pro‐inflammatory mediators and cytokines

**Figure 2:**
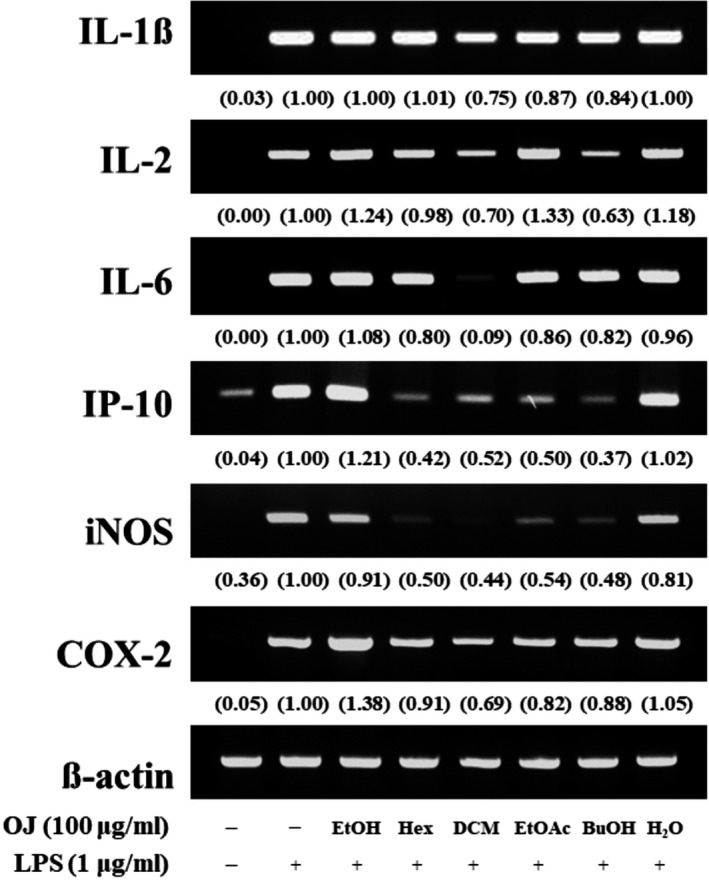
Inhibitory effect of the *Orostachys japonicus* solvent fractions on pro‐inflammatory mediators and cytokines. Macrophage cells were pretreated with solvent fractions (100 μg/ml) for 2 hr and LPS‐stimulated inflammation for 12 hr. Expression of iNOS, COX‐2, IL‐1β, IL‐2, IL‐6, and IP‐10 was analyzed by RT‐PCR

**Figure 3:**
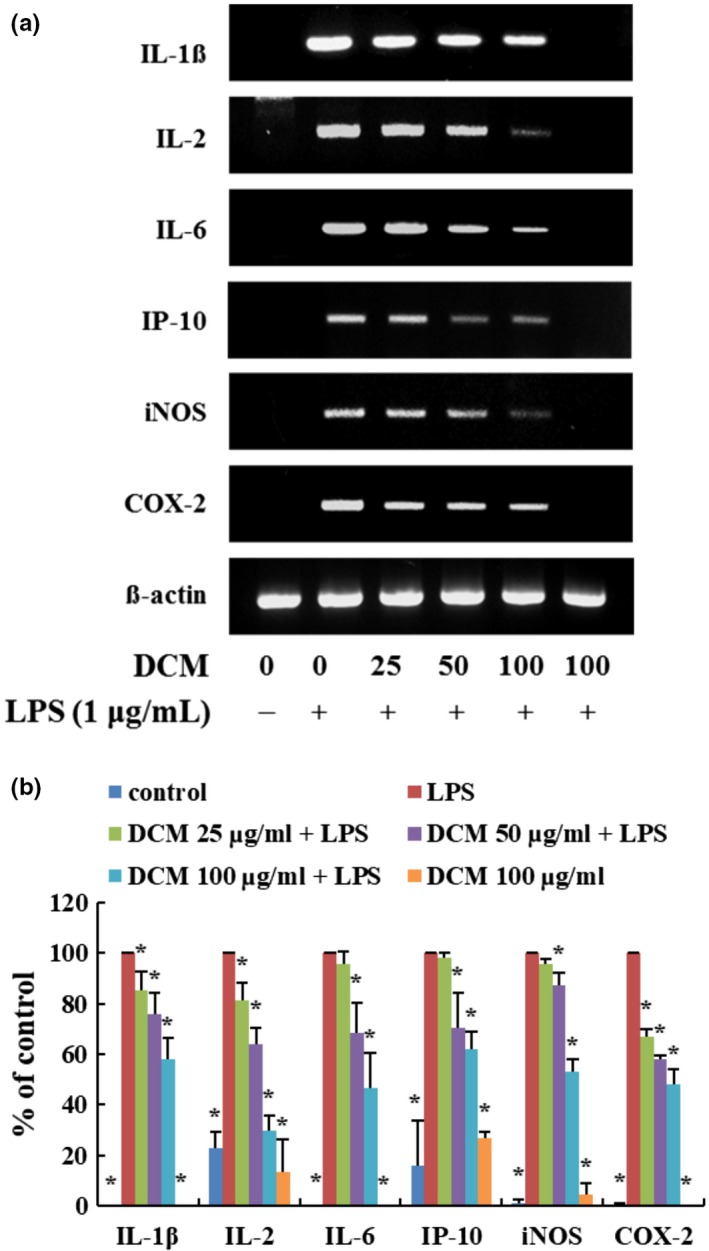
DCM fraction inhibits the pro‐inflammatory mediators and cytokine‐related mRNA levels. Macrophage cells were pretreated with DCM fraction at indicated concentrations for 2 hr and LPS‐stimulated inflammation for 12 hr. Expression of iNOS, COX‐2, IL‐1β, IL‐2, IL‐6, and IP‐10 was analyzed by RT‐PCR. Statistical significance is based on the difference when compared with only LPS‐treated cells (*p < .05)

The inflammatory response activates macrophages and pro‐inflammatory mediators, and cytokines are expressed, such as inducible nitric oxide synthase (iNOS), cyclooxygenase (COX)‐2, interleukin (IL)‐1β, IL‐2, IL‐6, and interferon‐gamma inducible protein 10 kDa (IP‐10). Their overproduction of these molecules is a common marker of the inflammatory response, resulting in various adverse effects (Park et al., 2017; Yao et al., 2016). Inhibiting the expression in these inflammatory mediators is important to verify the anti‐inflammatory activity of a natural substance. To investigate whether *O. japonicus* induces anti‐inflammatory mediators and cytokines, the expression of iNOS, COX‐2, IL‐1β, IL‐2, IL‐6, and IP‐10 was examined by RT‐PCR. The cells were pretreated to the organic solvent fractions (100 μg/ml) for 2 hr and LPS‐induced inflammation for 12 hr. As shown in Figure 2, the expressions of mRNA were decreased significantly in the DCM fraction, and we observed the expression of mRNA by the DCM fraction through serial concentrations. As shown in Figure 3, the expressions of mRNA were dramatically inhibited in a dose‐dependent manner. Therefore, this finding purposes that the DCM fraction had the most powerful effect on the mediators and cytokines caused by LPS‐stimulated inflammatory response.

### 3.3 Effect of the *Orostachys japonicus* solvent fractions on transcription factors

**Figure 4:**
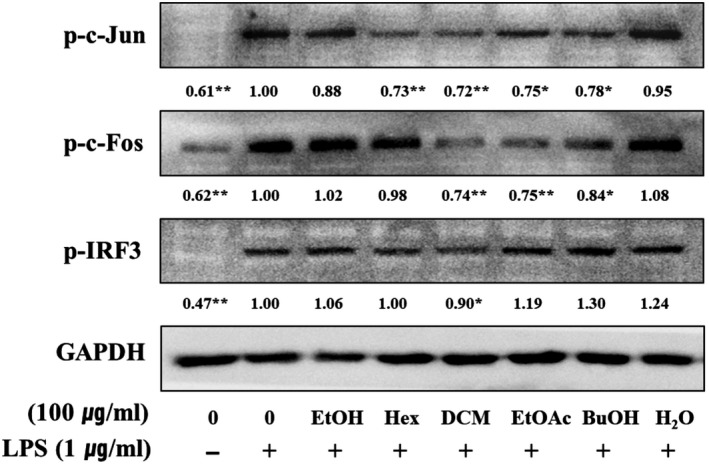
Inhibitory effect of the *Orostachys japonicus* solvent fractions on transcription factors. Macrophage cells were pretreated with solvent fractions (100 μg/ml) for 2 hr and LPS‐stimulated inflammation for 1–2 hr. Expression of phospho‐c‐Jun, phospho‐c‐Fos, and phospho‐IRF3 was analyzed by Western blotting. Macrophage cells were pretreated with DCM fraction at indicated concentrations for 2 hr and LPS‐stimulated inflammation for 12 hr. Expression of iNOS, COX‐2, IL‐1β, IL‐2, IL‐6, and IP‐10 was analyzed by RT‐PCR. Statistical significance is based on the difference when compared with only LPS‐treated cells (*p < .05 and **p < .01)

**Figure 5:**
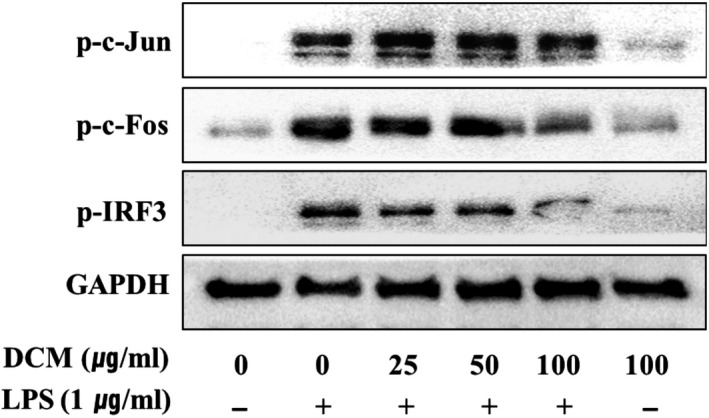
DCM fraction suppresses the transcription factor‐related protein levels. Macrophage cells were pretreated with DCM fraction at indicated concentrations for 2 hr and LPS‐stimulated inflammation for 1–2 hr. Expression of phospho‐c‐Jun, phospho‐c‐Fos, and phospho‐IRF3 was analyzed by Western blotting

Lipopolysaccharide binds TLR4, leading to the activation of intracellular signaling pathways of two types, such as the MyD‐88‐ and TRIF‐dependent pathways. Both of these pathways play important roles in regulating nuclear translocation of inflammatory factors, such as NF‐κB, AP‐1 (p‐c‐Jun and p‐c‐Fos), or IRF‐3. Pro‐inflammatory mediators and cytokines were expressed in LPS‐stimulated cells after the transcription factors are translocated to the nucleus. Inhibiting translocation into the nucleus is an important mechanism to regulate inflammation (Kim, Han, Kil, Seo, & Jin, 2019; Park et al., 2017; Takeda & Akira, 2005). To conform for inhibits of solvent fractions, cells were pretreated with the highest concentration of solvent fractions (100 μg/ml) for 2 hr and LPS‐induced inflammatory for 1.5 hr. As shown in Figure 4, the protein levels of AP‐1 (p‐c‐Jun and p‐c‐Fos) and p‐IRF‐3 were the lowest in the DCM fraction. We also detected a significant decrease in a dose‐dependent manner (Figure 5). These observations suggest that the DCM fraction is a negative regulator of LPS‐induced nuclear translocation of AP‐1 and IRF‐3 in macrophage cells. AP‐1 and IRF‐3 are regarded as pivotal factors in the regulation of inflammation by producing pro‐inflammatory mediators and cytokines, such as iNOS, COX‐2, IL‐1β, IL‐2, IL‐6, and IP‐10.

## 4 CONCLUSION

**Figure 6:**
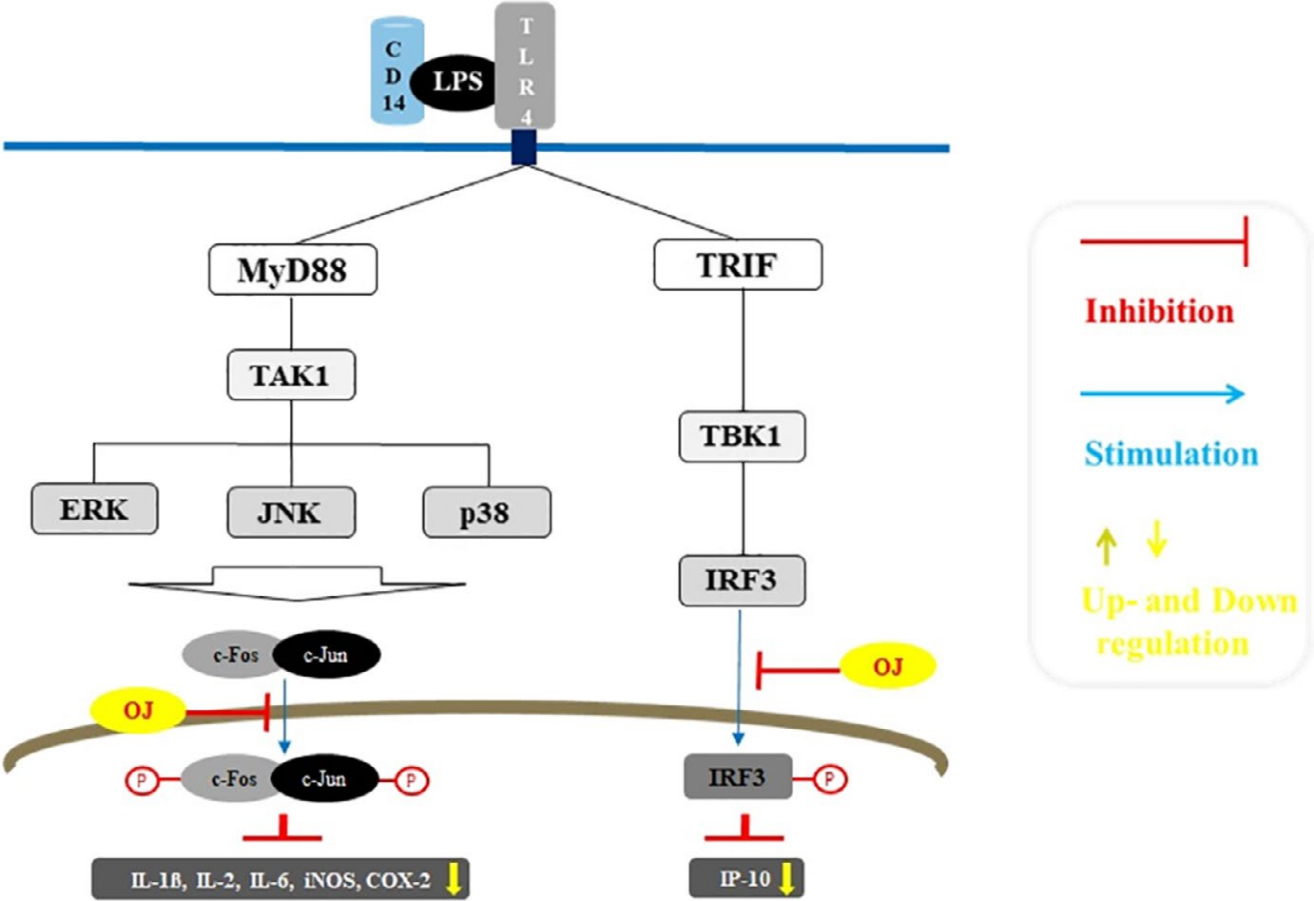
Anti‐inflammatory mechanisms of *Orostachys japonicus*

In conclusion, *O. japonicus* protected against the inflammatory response by reducing the nuclear translocation of inflammatory factors in LPS‐stimulated RAW264.7 macrophage cells. The present results demonstrated that the DCM fraction had an effect on the inhibitory activation on iNOS and COX‐2 under an inflammatory condition. Furthermore, the DCM fraction markedly attenuated the mRNA levels of pro‐inflammatory cytokines, including IL‐1β, IL‐2, IL‐6, and IP‐10. The transcription of AP‐1 and IRF‐3 activated along the MyD88‐ and TRIF‐dependent pathway was suppressed by the DCM fraction (Figure 6). Consequently, DCM fraction of organic solvents from *O. japonicus* is thought to have a strong anti‐inflammatory activity in LPS‐response cells.

## CONFLICT OF INTEREST

The author declares no conflict of interest.

## ETHICAL APPROVAL

This study does not involve any human or vertebrate animal.
